# Sex differences in leukocyte profile in ST-elevation myocardial infarction patients

**DOI:** 10.1038/s41598-020-63185-3

**Published:** 2020-04-22

**Authors:** Irene V. van Blokland, Hilde E. Groot, Tom Hendriks, Solmaz Assa, Pim van der Harst

**Affiliations:** University of Groningen, University Medical Center Groningen, Department of Cardiology, Groningen, The Netherlands

**Keywords:** Cardiovascular biology, Acute coronary syndromes

## Abstract

Background: Whether sex differences exist in the inflammatory response after ST-elevation myocardial infarction (STEMI) remains to be elucidated. We studied leukocyte profiles and their prognostic value in men and women presenting with STEMI. Methods: From a total of 552 consecutive STEMI patients, blood samples were collected at hospital admission. Linear regression was used to assess the relationship between leukocyte profiles and enzymatic infarct size. Cox regression was used to assess the association between leukocyte profiles and one-year mortality. Results: Women presented with higher lymphocyte counts (2.3·10^9^ cells/L (IQR 1.6–3.1) vs. 1.8·10^9^ cells/L (IQR 1.4–2.5), p = 3.00 ∙ 10^−4^) and percentages (21.1% (IQR 14.4–28.1) vs. 17.1% (IQR 12.3–24.3), p = 0.004). Lymphocyte to monocyte ratio (LMR) was also higher in women (3.25 (IQR 2.56–4.5) vs. 2.68 (IQR 2.08–3.59), p = 7.28 ∙ 10^−7^). Higher LMR was associated with lower peak CK-MB (β = −0.27 (95% CI: −0.50, −0.03), p = 0.026), lower peak troponin T (β = −0.45 (95% CI: −0.77, −0.13), p = 0.006) and lower one-year mortality risk (HR 0.35 (95% CI: 0.13, 0.96), p = 0.042). Conclusion: At admission for STEMI, women present with higher lymphocyte count and LMR. Higher LMR is associated with smaller infarct size and decreased one-year mortality risk and could be used as a biomarker to predict outcome.

## Introduction

Even though our knowledge of the pathophysiology of acute ST-elevation myocardial infarction (STEMI) has substantially increased over the past decades and inflammation showed to be an important player, the inflammatory mechanisms after STEMI remain, in part, to be determined.

In the inflammatory response after STEMI, apoptotic cardiomyocytes attract inflammatory cells to the ischemic tissue initiating cardiac repair and remodelling^1^. In the early stages, these inflammatory cells mostly consist of neutrophils while mononuclear cells as macrophages dominate as the inflammation progresses. Concurrently, the inflammatory cells secrete a variety of interleukins, which activate cardiac fibroblasts and initiate scar tissue formation^2–4^. Excess scar tissue may impair cardiac function, leading to an increased risk of developing heart failure^3^. Limiting scar tissue formation could therefore theoretically aid in reducing long-term consequences of the acute inflammatory response.

The extent of the immune response may be determined by the number and type of circulating immune cells^5^. The inflammatory response differs substantially between sexes where women are known to mount stronger innate and adaptive immune response compared to men^6^. This variation may be caused by steroid hormones, which are known to affect survival and differentiation of immune cells^5^. In general, oestrogens serve both pro- and anti-inflammatory roles^7^, whilst androgens have mainly anti-inflammatory effects^6,8^. Taking this into consideration, we might expect sex-specific differences in leukocyte profiles in STEMI patients. An improved understanding of these differences may aid in optimising sex-specific treatment of STEMI patients and improve risk stratification.

Recently, inflammation has been investigated in regard to the prognosis of STEMI patients^9^. The lymphocyte to monocyte ratio (LMR) has been regarded as a marker of inflammation which is related to coronary artery disease^10,11^. A lower LMR is thought to predict a worse prognosis as it is linked to higher mortality after STEMI. Furthermore, a lower LMR has recently been shown to be associated with a higher risk of having no-reflow after percutaneous coronary intervention (PCI) in STEMI patients^12^. In this study, we aimed to study differences between leukocyte profiles in men and women presenting with STEMI. We furthermore aimed to identify potential determinants of leukocyte profiles and study the prognostic value of LMR after STEMI in both sexes.

## Results

### Baseline characteristics

Baseline characteristics for women and men are shown in Table 1. Of the total population, 27% were women and the average age was 67 ± 12 years in women and 63 ± 12 years in men. The prevalence of cardiovascular risk factors was not significantly different between sexes, except for smoking habits (p = 0.001). Half of the women had hypertension, and 39% had hyperlipidemia. Previous stroke was more common in men (5% vs. 2%, p = 0.045). Women presented more often with a right coronary artery stenosis than men (51% vs. 38%, p = 0.004), whereas the left anterior descending artery (LAD) was more commonly the culprit vessel in men (41% vs 35%, p = 0.004). Women presented with higher levels of high sensitivity C-reactive protein (hsCRP) (3.4 mg/L, interquartile range (IQR) 1.6–7.8 vs. 2.2 mg/L, IQR 1.1–4.7, p = 0.004) and higher levels of N-terminal pro brain natriuretic peptide (NT-proBNP) (191 U/L, IQR 84–727 vs. 109 U/L, IQR 48–311, p = 3.78 ∙ 10^−6^). Furthermore, women had higher levels of total cholesterol (5.3 mmol/L, IQR 4.4–6.0, vs. 4.9 mmol/L, IQR 4.3–5.7, p = 0.012) and higher levels of high density lipoprotein (HDL) cholesterol (1.3 mmol/L, IQR 1.1–1.6, vs. 1.1 mmol/L, IQR 0.9–1.3, p = 1.24 ∙ 10^−11^). Peak values of CK, CK-MB and Troponin T did not differ between sexes. Medication use at admission was also similar between men and women (Supplementary Table 1).

**Table 1:** Baseline characteristics CardioLines population.

Characteristic	Women	Men	*P* value
Total	147 (27)	405 (73)	
Age, years	67 (12)	63 (12)	2.0 ∙ 10^–4^
BMI, kg/m^2^	26.5 (5.1)	27.2 (3.6)	0.110
**Blood pressure, mmHg**			
Systolic	139.7 (27.8)	136 (23.6)	0.088
Diastolic	80.1 (14.8)	83.9 (15.5)	0.011
Heart rate, bpm	77 (18)	75 (18)	0.190
**Cardiovascular risk factors**			
Smoking			0.001
Current smoker	65 (48%)	176 (46%)	
Former smoker	12 (9%)	86 (23%)	
Non-smoker	59 (43%)	119 (31%)	
Hypertension	66 (47%)	153 (40%)	0.158
Hyperlipidemia	54 (39%)	128 (34%)	0.320
Diabetes	61 (15%)	27 (19%)	0.360
Family history	146 (42%)	51 (42%)	0.920
Stroke	7 (2%)	7 (5%)	0.045
Peripheral artery disease	14 (4%)	5 (3%)	0.980
Ischemia time			0.500
<2 hours	31 (23%)	94 (26%)	
2–24 hours	93 (69%)	220 (62%)	
24–72 hours	2 (2%)	11 (3%)	
>72 hours	2 (2%)	7 (2%)	
Unknown	6 (4%)	25 (7%)	
Multi vessel disease			0.330
1 VD	58 (40%)	154 (38%)	
2 VD	36 (24%)	128 (32%)	
3 VD	45 (31%)	106 (26%)	
No significant stenoses	8 (5%)	15 (4%)	
**Infarct-related artery TIMI flow grade**			
Before intervention			0.860
0	197 (56%)	67 (52%)	
1	27 (7%)	9 (7%)	
2	50 (14%)	21 (16%)	
3	81 (23%)	32 (25%)	
After intervention			0.430
0	6 (2%)	4 (3%)	
1	5 (1%)	0 (0%)	
2	23 (7%)	9 (7%)	
3	319 (90%)	116 (90%)	
Culprit vessel			0.004
LAD	49 (35%)	152 (41%)	
RCA	71 (51%)	141 (38%)	
LCX	11 (8%)	66 (18%)	
Other	8 (5%)	15 (4%)	
Primary PCI	118 (80%)	339 (84%)	0.345
Thrombolytic therapy	0 (0%)	0 (0%)	NA
**Laboratory values**			
HbA1c, mmol/L	5.8 (5.5, 6.2)	5.7 (5.4, 6.0)	0.002
CRP, mg/L	3.4 (1.6, 7.8)	2.2 (1.1, 4.7)	0.004
Total cholesterol, mmol/L	5.3 (4.4–6.0)	4.9 (4.3–5.7)	0.012
LDL-cholesterol, mmol/L	3.5 (3.0–4.2)	3.5 (2.8–4.3)	0.670
HDL-cholesterol, mmol/L	1.3 (1.1, 1.6)	1.1 (0.9, 1.3)	1.235 ∙ 10^–11^
Triglycerides, mmol/L	0.9 (0.6–1.3)	0.9 (0.6–1.5)	0.096
NT-proBNP, U/L	191 (84, 727)	109 (48, 311)	3.782 ∙ 10^–6^
**Peak values**			
Peak CK, U/L	1042.5 (475, 1929)	1150 (464, 2772)	0.130
Peak CK-MB, U/L	130 (61.5, 243)	130.5 (51, 255)	0.670
Peak Troponin T, U/L	2376 (647, 5198)	2427 (827, 5951)	0.340

Data is expressed as number (%), as mean ± standard deviation (SD) for continuous variables with normal distributions and as median with inter-quartile range (IQR) for continuous variables with a skew distribution. BMI: Body Mass Index, CK: Creatine Kinase, CK-MB: Creatine kinase myocardial band, CRP: C-reactive protein, HbA1c: Glycated hemoglobin, HDL-cholesterol: High-density lipoprotein cholesterol, LDL-cholesterol: Low-density lipoprotein cholesterol, NT-proBNP: N-terminal pro brain natriuretic peptide, TIMI: Thrombosis In Myocardial Infarction, PCI: Percutaneous coronary intervention, VD: Vessel disease.

### Baseline levels of immune cell count in women and men post-STEMI

**Figure 1:**
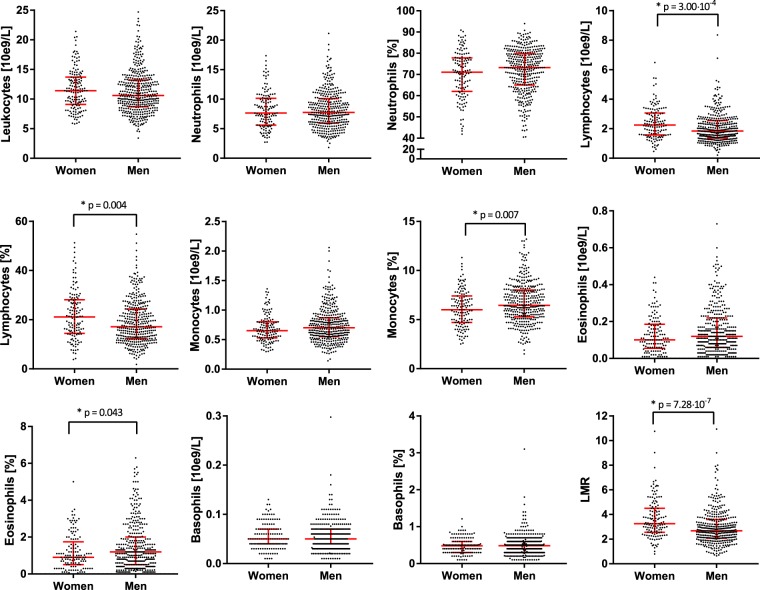
Leukocyte profile in ST-elevation myocardial infarction patients given per sex. Data is expressed as median with inter-quartile range (red error bars). Significant differences are expressed with a p-value. LMR: lymphocyte to monocyte ratio.

Women presented with higher lymphocyte count and percentages compared to men (2.3·10^9^cells/L, IQR 1.6–3.1 vs. 1.8·10^9^cells/L, IQR 1.4–2.5, p = 3.00 ∙ 10^−4^; 21.1%, IQR 14.4–28.1 vs. 17.1%, IQR 12.3–24.3, p = 0.004, respectively) (Fig. 1). LMR was also higher in women compared to men (3.25(IQR 2.56–4.5) vs. 2.68(IQR 2.08–3.59), p = 7.28 ∙ 10^–7^). Men presented with higher percentages of monocytes (6.5%, IQR 5.3–8.0 vs. 6.0%, IQR 4.7–7.4, p = 0.007) and eosinophils (1.2%, IQR 0.5–2.0 vs. 0.9%, IQR 0.5–1.6, p = 0.043).

### Predictors of immune cells

After multivariate adjustment, percentages of lymphocytes were independently associated with sex (β 0.16, 95% CI 0.05–0.27, p = 0.006), hsCRP (β −0.06, 95% CI −0.10–0.02, p = 0.002), HbA1c (β −2.29, 95% CI −4.43–0.14, p = 0.037) and triglycerides (β 0.14, 95% CI 0.06–0.22, p = 0.020) (Supplementary Table 2). Percentages of monocytes were independently associated with sex (β −0.89, 95% CI −1.39–0.40, p = 0.001), age (β 0.10, 95% CI 0.01–0.20, p = 0.043), triglycerides (β 0.36, 95% CI 0.04–0.68, p = 0.028), NT-proBNP (β 0.18, 95% CI 0.02–0.35, p = 0.028) and peak CK (β −0.22, 95% CI −0.39–0.06, p = 0.008). Percentages of eosinophils were independently associated with sex (β −0.26, 95% CI −0.48–0.04, p = 0.020), total cholesterol (β −0.09, 95% CI −0.17–0.01, p = 0.033) and triglycerides (β 0.27, 95% CI 0.13–0.42, p = 0.005). LMR was independently associated with sex (β 0.27, 95% CI 0.17–0.37, p = 3.82 ∙ 10^–6^), age (β −0.02, 95% CI −0.04 – 0.01, p = 0.035), systolic blood pressure (β 0.01, 95% CI 0.00–0.02, p = 0.022), hsCRP (β −0.05, 95% CI −0.09–0.01, p = 0.012), triglycerides (β 0.08, 95% CI 0.01–0.15, p = 0.032) and NT-proBNP (β −0.05, 95% CI −0.09–0.01, p = 0.006).

### Clinical outcomes

No significant differences were observed in outcomes at one-year follow-up between women and men (Table 2). A higher LMR was significantly associated with lower peak CK-MB (β −0.27, 95% CI −0.50–0.03, p = 0.026) and lower peak troponin T (β −0.45, 95% CI −0.77–0.13, p = 0.006), also after adjustment for age and sex (Table 3). A higher LMR was also significantly associated with a lower mortality risk at one-year (HR 0.35, 95% CI 0.13–0.96, p = 0.042). The association between LMR and re-ACS showed a similar trend, although not statistically significant. There were no interactions between LMR and sex on outcome variables, also after adjustment hs-CRP and NT-proBNP as proxy for ischemic time. ROC area for LMR on mortality at one year was 0.395. This did not differ from the ROC areas for monocytes and lymphocytes on mortality separately (p = 0.532) (Supplemental Fig. 1 and 2).

**Table 2:** Outcome at one year follow-up (CardioLines population).

Variable	Women (N = 147)	Men (N = 405)	*p* value
No reflow post PCI	4 (2.7%)	11 (2.7%)	1.000
Re PCI	6 (4.1%)	12 (3.0%)	0.930
Re ACS	7 (4.8%)	10 (2.5%)	0.053
Death	3 (2.0%)	14 (3.5%)	0.390

ACS: Acute coronary syndrome, PCI: Percutaneous coronary intervention.

**Table 3:** LMR as a predictor on outcome (CardioLines population).

Variable	Univariate regression			Multivariate regression*		
	Coef./OR/HR	95% CI	*P* value	Coef./OR/HR	95% CI	*P* value
Peak CK, log U/L	−0.19	−0.44–0.05	0.120	−0.17	−0.43–0.08	0.178
Peak CK-MB, log U/L	−0.02	−0.48–0.02	0.029	−0.27	−0.50–0.03	0.026
Peak Troponin T, log U/L	−0.51	−0.81–0.20	0.001	−0.45	−0.77–0.13	0.006
No TIMI reflow post-PCI	0.96	0.29–3.05	0.939	0.96	0.29–3.21	0.952
Re- PCI (1 year)	0.67	0.23–1.92	0.458	0.54	0.18–1.61	0.271
Re-ACS (1 year)	0.30	0.09–1.00	0.051	0.28	0.08–1.01	0.052
Death (1 year)	0.28	0.10–0.76	0.013	0.35	0.13–0.96	0.042

Coef: Coefficient, CI: Confidence interval, OR: Odds ratio, HR: Hazard ratio, ACS: Acute coronary syndrome, CK: Creatine kinase, CK-MB: Creatine kinase myocardial band, LMR: lymphocyte to monocyte ratio, PCI: Percutaneous coronary intervention. *After adjustment for age and sex.

### Reproducibility in GIPS III study

We validated our findings using data from the GIPS-III trial. In line with our results, levels of lymphocytes were higher in women (2.2 ·10^9^ cells/L, IQR 1.7·10^9–2.8^·10^9^ cells/L vs. 1.9·10^9^ cells/L, IQR 1.4–2.6 ·10^9^ cells/L, p = 0.14, respectively). Additionally, women had a higher LMR compared to men (4.1, IQR 3.4–5.5 vs. 3.1, IQR 2.2–4.4, p = 1.63 ∙ 10^–6^). Monocyte count and percentage of monocytes were both increased in men (0.6·10^9^ cells/L, IQR 0.5–0.8·10^9^ cells/L vs. 0.5·10^9^ cells/L, IQR 0.5–0.8·10^9^ cells/L, p = 0.005; 6.0%, IQR 4.9–7.3% vs. 5.3%, IQR 4.3–6.3%, p = 5.00 ∙ 10^–4^, respectively) (Supplementary Table 3).

After multivariate adjustment, sex remained significantly associated with lymphocyte count (β 0.24, 95% CI 0.13–0.35, p = 3.14 ∙ 10^–5^), monocyte count (β −0.14, 95% CI −0.25–0.03, p = 0.016), percentage of monocytes (β −0.18, 95% CI −0.26–0.09, p = 9.40 ∙ 10^–5^), and LMR (β 0.39, 95% CI 0.26–0.53, p = 1.01 ∙ 10^–8^) (Supplementary Table 4).

There were no significant differences in outcome at one-year follow-up between women and men (Supplementary Table 5). A higher LMR was significantly associated with lower peak CK (β −0.31, 95% CI −0.60–0.03, p = 0.032) and lower peak CK-MB (β −0.27, 95% CI −0.53–0.01, p = 0.039), also after adjustment for age and sex (Supplementary Table 6). A trend was observed between higher LMR and decreased risk of re-ACS (HR 0.28, 95% CI 0.06–1.26, p = 0.098). In addition, after adjustment for age and sex, higher LMR was associated with smaller infarct size (β −0.026, 95% CI −0.05–0.01, p = 0.025). There was no association between LMR and LVEF. We did not observe interactions with sex on infarct size (biochemical and functional) or outcomes at one-year follow-up.

## Discussion

In this study of 552 patients presenting with STEMI, we were able to provide an elegant overview of the leukocyte profiles in women and men and observed sex differences. Women had higher lymphocyte counts, lymphocyte percentages, and a higher LMR, whereas men presented with higher percentages of monocytes and eosinophils. A higher LMR was associated with smaller infarct size and a lower mortality risk at one-year. These effects did not result in different outcomes between women and men.

The observed differences in activated lymphocytes, monocytes and eosinophils support previous research, where it has been shown that, after *in vitro* stimulation of peripheral blood mononuclear cells (PBMCs), women presented with higher numbers of activated and proliferating T-cells and B-cells in peripheral blood^13,14^. Furthermore, B-cell activation was higher in women measured by higher antibody responses, higher basal immunoglobulin levels and higher numbers of B-cells^13^. Sex hormones might play an important role in these observations as both T- and B-cells are known to have oestrogen receptors^15^. In line to this, hormonal depletion of androgens in male mice has shown to lead to higher numbers of T cells, suggesting a negative effect of male hormones on lymphocytes^16^.

We observed increased levels of monocytes in men. *In vitro* studies have shown that the number and function of monocytes in whole blood was higher in men versus women, after stimulation with lipopolysaccharide (LPS)^17^. The exact underlying mechanism has not been elucidated yet, but it has been suggested that monocytes in men are in a higher state of excitation compared to women^17^.

Limited is known about sex differences in eosinophils in STEMI patients. Previous research studied eosinophil levels in 620 STEMI patients and observed an association between a decrease in eosinophils and higher risk of major adverse events^18^. However, analyses were not stratified by sex. It is known that oestrogen regulates eosinophil kinetics. In female mice greater eosinophilic infiltration into lung tissues has been observed, but the exact mechanisms underlying and whether this phenomenon is also present in post-menopausal women remains to be elucidated^19^.

Recently, it has been shown that leukocyte profiles differ across different ages in patients presenting with a STEMI^20^. Elderly STEMI patients presented with a higher acute pro-inflammatory profile (i.e. higher leukocytes and neutrophil-to-lymphocyte ratio) compared to young patients. Furthermore, an increased inflammatory response was associated with no-reflow and higher mortality post-STEMI. Another study showed that women younger than 71 years have a higher one-year mortality compared to the older women, whereas older women had a lower mortality compared to older men^21^. Our study is of additive value, since it provides a comprehensive overview of the leukocyte profiles directly after MI and their potential associations with outcome. Furthermore, we were able to reproduce our findings in a separate STEMI cohort of 379 patients and observed similar results. It is therefore of interest to further elucidate the interplay between the immune system, sex and age in STEMI patients.

In our study we showed that higher LMR was associated with smaller infarct size (biochemical and functional) and lower mortality. This is in accordance with previous literature, which found that a low LMR at 48 hours after admission was independently associated with both an increased short- and long-term mortality in STEMI patients^22^. It has been hypothesized that the association of lower LMR with higher mortality is due to an elevation of serum catecholamines and cortisol levels during a systemic stress response, resulting in lymphocyte apoptosis. This reflects a suppressed immune system. Others speculate on down-regulation of lymphocyte proliferation and differentiation, and lymphocyte redistribution within the lymphopoietic systems^22^.

Until now it is unknown whether the difference in LMR between women and men is also associated with different prognosis in both sexes. One would hypothesize that women have lower rates of re-events or mortality, since they have a higher LMR. In our study we did not observe different outcomes after one year between women and men. There was also no significant interaction between sex and LMR on clinical outcomes. Women, however, had higher CRP and NT-proBNP levels at baseline, which possibly reflects a longer ischemia time. Although the reported ischemia time was not different between men and women, there may be a discrepancy between real ischemia time and the one that is reported, as women with STEMI more often present with atypical symptoms which are not immediately recognized as ACS^23^. It is possible that such differences have partly affected the association of LMR with clinical outcomes. However, after adjustment for ischemic time, hs-CRP and NT-proBNP, we still did not observe an interaction between LMR and sex on clinical outcome. Contrary to the categorical variable of ischemic time in the CardioLines population, we were able to investigate ischemic time more precisely in the GIPS-III study. Women had a slightly longer ischemic time (177 minutes vs. 156 minutes), but this difference was not significant. Similarly, in the GIPS-III cohort, we did not find an interaction between LMR and sex on clinical outcomes either.

Another explanation could be the small number of events. Further research in a larger STEMI population is warranted to investigate the possible interaction between LMR and sex on clinical outcomes.

This study possess some limitations. First, this was a single-center study with very low adverse event rate during the follow-up period. However, the already observed association between LMR and re-ACS invites to further investigate this association in a larger population. Second, we only had information on the quantity of the cells. It would be of value to also possess information on the functionality and activity of cells, and cytokine levels. Nevertheless, this study could serve as a starting point, providing new insights where to focus concerning new possible therapeutic targets.

Our study shows that women presented with higher lymphocyte count and LMR, whereas men had higher percentages of monocytes and eosinophils after a STEMI. A higher LMR was associated with smaller infarct size and a decreased mortality risk at one-year. However, this association was not different between sexes. Further research in a larger STEMI population is needed to investigate the possible interaction between leukocyte profile and sex on clinical outcomes.

## Methods

### Study population and design

The CardioLines biobank is a single-center, observational biobank aimed to study potential factors related to success or failure of diagnosis and treatment both from a patient as well as from a medical perspective^24^. The study protocol was in accordance with the Declaration of Helsinki and was approved by the local ethics committee (Groningen, the Netherlands) and national regulatory authorities. All methods were performed in accordance with the relevant guidelines and regulations. The current study is a substudy of the CardioLines biobank, including all STEMI patients with available baseline data and leukocyte profiling at admission between July 3^rd^, 2015, and November 23^rd^, 2017 (N = 552). Inclusion criteria were presentation with ST-elevation myocardial infarction (STEMI), according to the European Society of Cardiology guideline^25^, and age of 18 years or higher. Due to the acute nature of myocardial infarction, all participants included in the study initially provided verbal consent, and written informed consent was obtained at a later stage. Blood samples were collected at cardiac catheterization (T = 0). In case written informed consent could not be obtained due to early mortality, subjects were included by deferred consent. Ischemic time was defined as the time measured in hours from onset of complaints until cardiac catheterization. Variables regarded as prognostic outcomes after STEMI were and peak values of CK, CK-MB and Troponin T, no reflow post-PCI, re-PCI (defined as recurrent coronary intervention) within one-year after STEMI, re-ACS (defined as instable angina pectoris, non ST-segment elevation myocardial infarction, or STEMI) within one-year after STEMI, and death within one-year after STEMI. The definitions used have been described earlier^26^. No reflow post-PCI was defined as a Thrombosis In Myocardial Infarction (TIMI) grade of 0 and 1. In order to test the reproducibility of our findings, data from the glycometabolic intervention as adjunct to primary percutaneous intervention in ST elevation myocardial infarction (GIPS-III) trial was used. This study was designed to evaluate the effect of metformin treatment on left ventricular function in 379 STEMI patients without known diabetes. Details on the design of the GIPS-III trial have been reported previously^27^.

### Data collection

At admission, standard laboratory assessment was performed and standard physical examination parameters were measured according to protocol. LMR was computed as the lymphocyte count to monocyte count measured at admission. Clinical follow-up was obtained from the central personal records database and hospital records, and if not available, with telephone contact..

### Statistical analysis

Continuous variables with a normal distribution were summarized as mean ± standard deviation and continuous variables with a skewed distribution were presented as median with interquartile ranges. Discrete variables were presented as percentages. To compare groups between continuous normally distributed variables we used Student T-tests and the Mann-Whitney U test for skewed continuous variables. The Chi-square and Fisher exact test were used to compare groups between categorical variables. Univariate and multivariate linear and logistic regression analysis were applied to study associations between cell count and possible predictors. Cell counts significantly differing between both sexes at baseline were taken for univariate regression with covariates. If skewed, variables were log transformed. Age, sex, and variables with a *P* value less than 0.2 were included in multivariate regression analysis. Univariate linear and logistic regression analyses were performed to evaluate the association between LMR and peak CK, peak CK-MB, peak troponin T, and no reflow post-PCI. A Cox proportional hazard regression analysis was performed to study the association between LMR and re-PCI, re-ACS, and mortality at one year. We calculated receiver operating characteristic (ROC) curves for LMR, lymphocytes and monocytes on mortality at one year, using the De Long method. We also tested for interaction with sex. In the GIPS-III trial cardiac function has been determined by magnetic resonance imaging at 4 months after MI. We tested whether LMR was associated with cardiac function. A two-sided *P* value less than 0.05 was considered statistically significant for all analyses. Statistical analysis was performed using Stata version 15.0 (StataCorp, College Station, Texas, USA).

## Supplementary information

Supplementary Information.

Supplementary Information2.

Supplementary Information3.

## Data Availability

Data can be made accessible on request
