# Supplementary figures and images for: Sex differences in leukocyte profile in ST-elevation myocardial infarction patients
Source: Sci Rep. 2020 Apr 22;10:6851. doi: 10.1038/s41598-020-63185-3 (PMC7176674; doi:10.1038/s41598-020-63185-3)

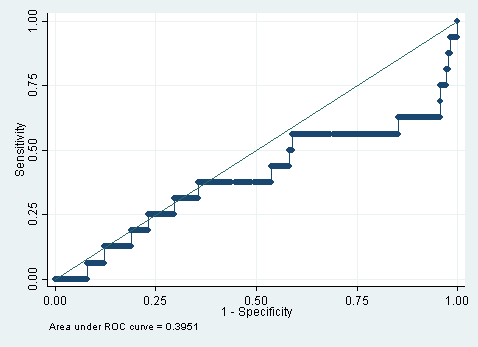

Supplement: Supplementary file 1 — Supplementary Information. [file 41598_2020_63185_MOESM1_ESM.tif]

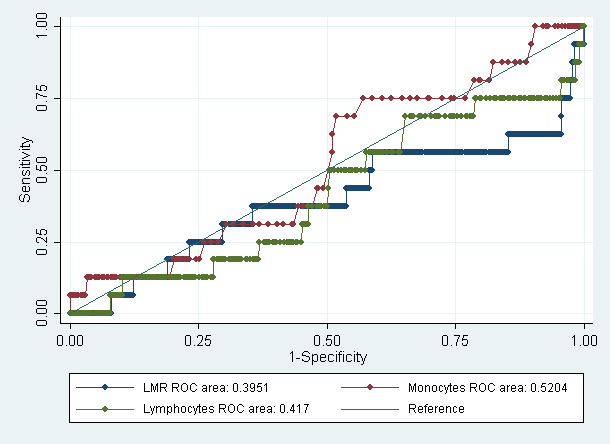

Supplement: Supplementary file 2 — Supplementary Information2. [file 41598_2020_63185_MOESM2_ESM.tif]
