# Supplementary material for: Sex differences in leukocyte profile in ST-elevation myocardial infarction patients
Source: Sci Rep. 2020 Apr 22;10:6851. doi: 10.1038/s41598-020-63185-3 (PMC7176674; doi:10.1038/s41598-020-63185-3)
Supplement: Supplementary file 3 — Supplementary Information3. [file 41598_2020_63185_MOESM3_ESM.docx]

**Supplementary material**

**Sex differences in leukocyte profile in ST-elevation myocardial infarction patients**

Irene V. van Blokland, B.Sc.; Hilde E. Groot, M.D.; Tom Hendriks, M.D.; Solmaz Assa, M.D., Ph.D.; Pim van der Harst, M.D. Ph.D.

**Supplementary Table 1. Medication use at admission (CardioLines population).**

| Characteristic | Women | Men | *P* value |
| --- | --- | --- | --- |
| B-blocker | 75 (19%) | 31 (22%) | 0.450 |
| ACEi | 53 (14%) | 22 (16%) | 0.540 |
| AT-II antagonist | 42 (10%) | 18 (13%) | 0.490 |
| Potassium sparing diuretic | 6 (2%) | 1 (1%) | 0.470 |
| Loop diuretic | 6 (2%) | 6 (4%) | 0.060 |
| Thiazide diuretic | 39 (10%) | 23 (17%) | 0.041 |
| Ascal | 62 (16%) | 21 (15%) | 0.840 |
| Clopidogrel | 9 (2%) | 1 (1%) | 0.240 |
| Ticagrelor | 7 (2%) | 1 (1%) | 0.370 |
| LMWH | 1 (0.3%) | 1 (1%) | 0.440 |
| Oral anticoagulant | 16 (4%) | 6 (4%) | 0.910 |
| Statin | 70 (18%) | 26 (19%) | 0.850 |
| Insulin | 11 (3%) | 12 (9%) | 0.004 |
| Oral antidiabetic | 22 (6%) | 14 (10%) | 0.076 |
| PPI | 88 (23%) | 38 (28%) | 0.250 |

Data is expressed as number (frequency %). ACEi: Angiotensin-converting-enzyme inhibitor, AT-II antagonist: Angiotension II antagonist, Ascal: Carbasalate calcium, B-blocker: Beta-blocker, LMWH: Low-molecular weight heparine, PPI: Proton pump inhibitor.

**Supplementary Table 2. Predictors of leukocytes in univariate and multivariate regression analyses in STEMI patients (CardioLines population).**

| Variable | Univariate analysis | | | | | | Multivariate analysis | | | |  | |  |
| --- | --- | --- | --- | --- | --- | --- | --- | --- | --- | --- | --- | --- | --- |
| **Lymphocytes (%)** | | **Coef.** | | **95% CI** | | ***P* value** | | **Coef.** | | **95% CI** | | ***P* value** | |
| Sex (woman) | | 0.14 | | 0.04 - 0.23 | | 0.006 | | 0.16 | | 0.05 - 0.27 | | 0.006 | |
| Age (per 5 years) | | -0.01 | | -0.02 - 0.01 | | 0.654 | | -0.00 | | -0.01 - 0.00 | | 0.790 | |
| Systolic blood pressure (per 5 mmHg) | | 0.01 | | -0.01 - 0.02 | | 0.122 | | 0.00 | | -0.00 - 0.00 | | 0.165 | |
| Heart rate (per 5 bpm) | | -0.01 | | -0.02 - 0.00 | | 0.043 | | -0.00 | | -0.00 - 0.00 | | 0.586 | |
| Culprit vessel (RCA vs. LAD) | | 0.09 | | -0.02 – 0.19 | | 0.093 | | -0.01 | | -0.11 – 0.10 | | 0.947 | |
| Insulin | | 0.29 | | 0.05 - 0.52 | | 0.016 | | 0.06 | | -0.20 - 0.32 | | 0.642 | |
| CRP (per log mg/L) | | -0.06 | | -0.10 - -0.03 | | 0.001 | | -0.06 | | -0.10 - -0.02 | | 0.002 | |
| Hba1c (per inverse mmol/L) | | -2.09 | | -4.08 - -0.09 | | 0.041 | | -2.29 | | -4.43 - -0.14 | | 0.037 | |
| HDL (per log mmol/L) | | -0.11 | | -0.26 - 0.03 | | 0.129 | | -0.07 | | -0.26 - 0.12 | | 0.457 | |
| Triglycerides (per log) | | 0.15 | | 0.08 - 0.22 | | 4.30∙10^-5^ | | 0.14 | | 0.06 - 0.22 | | 0.020 | |
| NTproBNP (per log U/L) | | -0.06 | | -0.09 - -0.03 | | 1.51∙10^-4^ | | -0.02 | | -0.06 - 0.01 | | 0.218 | |
| Peak CK (per log U/L) | | -0.07 | | -0.11 - -0.03 | | 2.34∙10^-4^ | | -0.04 | | -0.08 - 0.01 | | 0.119 | |
| **Monocytes (%)** | | **Coef.** | | **95% CI** | | ***P* value** | | **Coef.** | | **95% CI** | | ***P* value** | |
| Sex (woman) | | -0.58 | | -0.98 - -0.18 | | 0.005 | | -0.89 | | -1.39 - -0.40 | | 0.001 | |
| Age (per 5 years) | | 0.13 | | 0.05 - 0.20 | | 0.001 | | 0.10 | | 0.01 - 0.20 | | 0.043 | |
| Diastolic blood pressure (per 5 mmHg) | | -0.04 | | -0.10 - 0.02 | | 0.146 | | -0.05 | | -0.12 - 0.02 | | 0.167 | |
| Heart rate (per 5 bpm) | | -0.04 | | -0.10 - 0.01 | | 0.092 | | -0.02 | | -0.08 - 0.04 | | 0.470 | |
| Hypertension | | 0.36 | | -0.00 - 0.73 | | 0.053 | | 0.24 | | -0.16 - 0.64 | | 0.246 | |
| Insulin | | 0.75 | | -0.20 - 1.71 | | 0.121 | | -0.36 | | -1.50 - 0.78 | | 0.535 | |
| Oral antidiabetic | | 0.54 | | -0.20 - 1.28 | | 0.153 | | 0.04 | | -0.83 - 0.91 | | 0.924 | |
| Hba1c (per inverse mmol/L) | | -7.96 | | -16.17 - 0.25 | | 0.057 | | -7.83 | | -18.50 - 2.83 | | 0.149 | |
| Total cholesterol (per log ) | | -0.23 | | -0.40 - -0.07 | | 0.004 | | -0.05 | | -0.26 - 0.16 | | 0.646 | |
| HDL (per log mmol/L) | | -0.43 | | -1.03 - 0.17 | | 0.156 | | 0.17 | | -0.67 - 1.00 | | 0.697 | |
| Triglycerides (per log) | | 0.24 | | -0.05 - 0.52 | | 0.111 | | 0.36 | | 0.04 - 0.68 | | 0.028 | |
| NTproBNP (per log U/L) | | 0.02 | | 0.02 - 0.27 | | 0.023 | | 0.18 | | 0.02 - 0.35 | | 0.028 | |
| Peak CK (per log U/L) | | -0.25 | | -0.41 - -0.09 | | 0.003 | | -0.22 | | -0.39 - -0.06 | | 0.008 | |
| **Eosinophils (%)** | | **Coef.** | | **95% CI** | | ***P* value** | | **Coef.** | | **95% CI** | | ***P* value** | |
| Sex (woman) | | -0.19 | | -0.39 - 0.01 | | 0.064 | | -0.26 | | -0.48 - -0.04 | | 0.020 | |
| Age (per 5 years) | | 0.01 | | -0.03 – 0.05 | | 0.709 | | 0.01 | | -0.00 - 0.02 | | 0.065 | |
| Diastolic blood pressure (per 5 mmHg) | | -0.02 | | -0.05 – 0.00 | | 0.095 | | -0.01 | | -0.03 - 0.03 | | 0.979 | |
| Heart rate (per 5 bpm) | | -0.03 | | -0.06 - -0.01 | | 0.012 | | -0.01 | | -0.04 - 0.02 | | 0.508 | |
| CRP (per log mg/L) | | -0.07 | | -0.14 – 0.00 | | 0.066 | | -0.04 | | -0.11 - 0.04 | | 0.361 | |
| Total cholesterol (per log mmol/L) | | -0.05 | | -0.13 – 0.03 | | 0.188 | | -0.09 | | -0.17 -0.01 | | 0.033 | |
| HDL (per log mmol/L) | | -0.46 | | -0.76 - -0.17 | | 0.002 | | -0.07 | | -0.45 - 0.31 | | 0.706 | |
| Triglycerides (per log mmol/L) | | 0.28 | | 0.14 – 0.42 | | 1.10∙10^-4^ | | 0.27 | | 0.13 - 0.42 | | 0.005 | |
| NTproBNP (per log U/L) | | -0.13 | | -0.19 - -0.07 | | 3.50∙10^-5^ | | -0.06 | | -0.13 - 0.02 | | 0.122 | |
| Peak CK (per log U/L) | | -0.15 | | -0.23 - -0.08 | | 1.31∙10^-4^ | | -0.05 | | -0.13 - 0.03 | | 0.260 | |
| **LMR** | | **Coef.** | | **95% CI** | | ***P* value** | | **Coef.** | | **95% CI** | | ***P* value** | |
| Sex (woman) | | 0.22 | | 0.14 – 0.31 | | 1.02∙10^-6^ | | 0.27 | | 0.17 – 0.37 | | 3.82∙10^-6^ | |
| Age (per 5 years) | | -0.02 | | -0.04 – 0.01 | | 0.008 | | -0.02 | | -0.04 – 0.01 | | 0.035 | |
| Systolic blood pressure (per 5 mmHg) | | 0.00 | | -0.01 – 0.00 | | 0.051 | | 0.01 | | 0.00 – 0.02 | | 0.022 | |
| Hypertension | | -0.06 | | -0.14 – 0.02 | | 0.161 | | -0.04 | | -0.13 – 0.04 | | 0.344 | |
| Insulin | | 0.15 | | -0.07 – 0.38 | | 0.170 | | 0.04 | | -0.18 – 0.26 | | 0.735 | |
| CRP (per log mg/L) | | -0.06 | | -0.10 - -0.03 | | 1.19∙10^-4^ | | -0.05 | | -0.09 - -0.01 | | 0.012 | |
| Total cholesterol (per log mmol/L) | | 0.04 | | 0.00 – 0.07 | | 0.057 | | -0.01 | | -0.05 – 0.03 | | 0.636 | |
| Triglycerides (per log) | | 0.09 | | 0.02 – 0.15 | | 0.008 | | 0.08 | | 0.01 – 0.15 | | 0.032 | |
| NTproBNP (per log U/L) | | -0.08 | | -0.11 - -0.05 | | 1.57∙10^-8^ | | -0.05 | | -0.09 - -0.01 | | 0.006 | |
| Peak CK (per log U/L) | | -0.03 | | -0.06 -0.01 | | 0.120 | | 0.00 | | -0.04 – 0.04 | | 0.853 | |
| **Lymphocytes (count)** | **Coef.** | | **95% CI** | | ***P* value** | | **Coef.** | | **95% CI** | | ***P* value** | |  |
| Sex (woman) | 0.16 | | 0.07 - 0.26 | | 0.001 | | 0.21 | | 0.10 - 0.32 | | 2.11∙10^-4^ | |  |
| Age (per 5 years) | -0.03 | | -0.05 - -0.02 | | 1.19∙10^-4^ | | -0.04 | | -0.06 - -0.02 | | 0.001 | |  |
| Smoking | 0.06 | | 0.00 - 0.12 | | 0.049 | | 0.05 | | -0.01 - 0.11 | | 0.086 | |  |
| Insulin | 0.21 | | -0.01 - 0.43 | | 0.056 | | -0.02 | | -0.28 - 0.24 | | 0.874 | |  |
| Hba1c (per inverse mmol/L) | -2.49 | | -4.38 - -0.59 | | 0.010 | | -2.53 | | -4.61 - -0.46 | | 0.017 | |  |
| HDL (per log mmol/L) | -0.15 | | -0.29 - -0.02 | | 0.030 | | -0.09 | | -0.27 - 0.08 | | 0.306 | |  |
| Triglycerides (per log) | 0.16 | | 0.09 - 0.22 | | 3.71∙10^-6^ | | 0.12 | | 0.04 - 0.19 | | 0.002 | |  |
| NTproBNP (per log U/L) | -0.04 | | -0.07 -0.02 | | 0.003 | | -0.00 | | -0.04 - 0.04 | | 0.994 | |  |
| Peak CK (per log U/L) | -0.04 | | -0.08 - 0.00 | | 0.050 | | -0.02 | | -0.06 - 0.02 | | 0.388 | |  |

Coef.: Coefficient, CI: Confidence interval, CK: Creatine kinase, CRP: C-reactive protein, HDL: High-density lipoprotein, HbA1c: Glycated hemoglobin, NT-proBNP: N-terminal pro brain natriuretic peptide.

Supplementary Table 3. Characteristics of participants of the GIPS-III trial

| Characteristic | Female | Male | *P* value |
| --- | --- | --- | --- |
| Total | 95 (25) | 284 (75) |  |
| Age, years | 60 (12) | 58 (12) | 0.110 |
| BMI, kg/m^2^ | 27.3 (4.7) | 26.8 (3.5) | 0.320 |
| Blood pressure, mmHg |  |  |  |
| Systolic | 137.0 (25.8) | 133.5 (22.5) | 0.210 |
| Diastolic | 82.6 (14.8) | 84.9 (14.5) | 0.180 |
| Heart rate, bpm | 74 (15) | 76 (17) | 0.310 |
| Active smoker at randomisation | 56 (59%) | 153 (54%) | 0.390 |
| Hypertension | 40 (42%) | 72 (25%) | 0.002 |
| Hypercholesterolemia | 58 (61%) | 181 (64%) | 0.640 |
| Stroke | 0 (0.0%) | 3 (1%) | 0.310 |
| Total ischemic time, minutes | 177.0 (109.0, 267.0) | 156.0 (109.0, 247.0) | 0.220 |
| Single vessel disease | 66 (70%) | 192 (68%) | 0.740 |
| Culprit vessel |  |  |  |
| LAD | 37 (39%) | 109 (38%) | 0.610 |
| LCX | 13 (14%) | 51 (18%) |  |
| RCA | 45 (47%) | 124 (44%) |  |
|  |  |  |  |
| TIMI flow |  |  |  |
| 0 | 57 (60%) | 151 (53%) | 0.640 |
| 1 | 5 (5%) | 22 (8%) |  |
| 2 | 16 (17%) | 50 (18%) |  |
| 3 | 17 (18%) | 61 (22%) |  |
| TIMI flow |  |  |  |
| 2 | 10 (11%) | 24 (9%) | 0.540 |
| 3 | 85 (90%) | 260 (92%) |  |
| Laboratory values |  |  |  |
| HbA1c, mmol/L  CRP, mg/L  Total cholesterol, mmol/L  HDL-cholesterol, mmol/L  LDL-cholesterol, mmol/L  Triglycerides, mmol/L  NT-proBNP, ng/L  Peak values  Peak CK, U/L  Peak CK-MB, U/L  Peak Troponin T, ng/L | 5.9 (5.6, 6.1)  2.8 (1.1, 6.5)  5.3 (4.8, 6.0)  1.3 (1.1, 1.5)  3.7 (3.2, 4.3)  0.8 (0.6, 1.0)  133.0 (66.0, 278.0)  1206.0 (391.0, 2940.0)  142.0 (50.0, 305.0)  2.6 (0.9, 5.6) | 5.8 (5.6, 6.0)  1.9 (0.9, 3.8)  5.3 (4.7, 6.0)  1.1 (0.9, 1.3)  3.8 (3.2, 4.5)  1.1 (0.7, 1.7)  67.5 (36.0, 161.5)  1360.5 (665.0, 3170.5)  163.0 (76.0, 335.5)  2.9 (1.2, 6.4) | 0.019  0.008  0.990  4.28∙10^-7^  0.450  2.37∙10^-6^  1.64∙10^-5^  0.230  0.370  0.480 |
| Leukocyte profile |  |  |  |
| Leukocyte count, 109 cells/L | 11.5 (8.9, 13.9) | 10.8 (8.7, 13.3) | 0.280 |
| Neutrophil count, 109 cells/L | 7.9 (5.3, 9.8) | 7.6 (5.4, 9.8) | 0.990 |
| Percentage of neutrophils, % | 70.7 (62.6, 78.2) | 73.6 (64.0, 80.4) | 0.340 |
| Lymphocyte count, 109 cells/L | 2.2 (1.7, 2.8) | 1.9 (1.4, 2.6) | 0.014 |
| Percentage of lymphocytes, % | 22.0 (15.3, 28.3) | 19.2 (12.6, 26.1) | 0.055 |
| Monocyte count, 109 cells/L | 0.5 (0.4, 0.7) | 0.6 (0.5, 0.8) | 0.005 |
| Percentage of monocytes, % | 5.3 (4.3, 6.3) | 6.0 (4.9, 7.3) | 5.00∙10^-4^ |
| Eosinophil count, 109 cells/L | 0.1 (0.1, 0.2) | 0.1 (0.1, 0.2) | 0.290 |
| Percentage of eosinophils, % | 1.1 (0.6, 1.8) | 1.2 (0.5, 2.1) | 0.350 |
| Basophil count, 109 cells/L | 0.0 (0.0, 0.0) | 0.0 (0.0, 0.0) | 0.400 |
| Percentage of basophils, % | 0.2 (0.2, 0.4) | 0.2 (0.2, 0.4) | 0.500 |
| LMR | 4.1 (3.4, 5.5) | 3.1 (2.2, 4.4) | 1.63∙10^-6^ |

Data is expressed as number (%), as mean ± standard deviation (SD) for continuous variables with normal distributions and as median with inter-quartile range (IQR) for continuous variables with a skew distribution. BMI: Body Mass Index, CK: Creatine Kinase, CK-MB: Creatine kinase myocardial band, CRP: C-reactive protein, HbA1c: Glycated hemoglobin, HDL-cholesterol: High-density lipoprotein cholesterol, LDL-cholesterol: Low-density lipoprotein cholesterol, NT-proBNP: N-terminal pro brain natriuretic peptide, TIMI: Thrombosis In Myocardial Infarction, LMR: lymphocyte to monocyte ratio

**Supplementary Table 4. Predictors of leukocytes in univariate and multivariate regression analyses in STEMI patients (GIPS III trial).**

| Variable | Univariate analysis | | | | Multivariate analysis | | |  | |  |
| --- | --- | --- | --- | --- | --- | --- | --- | --- | --- | --- |
| **Lymphocyte count** | | **Coef.** | **95% CI** | ***P* value** | | **Coef.** | **95% CI** | | ***P* value** | |
| Sex (woman) | | 0.16 | 0.04 - 0.27 | 0.007 | | 0.24 | 0.13 - 0.35 | | 3.14∙10^-5^ | |
| Age (per 5 years) | | -0.04 | -0.06 - 0.01 | 0.001 | | -0.03 | -0.05 - -0.01 | | 0.011 | |
| Hypertension | | 0.08 | -0.02 - 0.19 | 0.120 | | 0.08 | -0.02 - 0.02 | | 0.109 | |
| Hba1c (per inverse mmol/L) | | -4.27 | -7.84 - -0.71 | 0.019 | | -3.72 | -7.03 - -0.40 | | 0.028 | |
| HDL (per log mmol/L) | | -0.20 | -0.38 - -0.03 | 0.021 | | 0.06 | -0.13 - 0.26 | | 0.504 | |
| Triglycerides (per log) | | 0.23 | 0.15 - 0.30 | 1.01∙10^-8^ | | 0.22 | 0.14 - 0.31 | | 9.94∙10^-7^ | |
| NTproBNP (per log U/L) | | -0.06 | -0.09 - -0.02 | 0.003 | | -0.04 | -0.08 - 0.00 | | 0.038 | |
| **Monocyte count** | | **Coef.** | **95% CI** | ***P* value** | | **Coef.** | **95% CI** | | ***P* value** | |
| Sex (woman) | | -0.16 | -0.26 - -0.07 | 0.001 | | -0.14 | -0.25 - -0.03 | | 0.016 | |
| Age (per 5 years) | | -0.03 | -0.05 - -0.01 | 0.002 | | -0.20 | -0.04 - 0.00 | | 0.067 | |
| CRP (per log mg/L) | | 0.04 | 0.00 – 0.09 | 0.051 | | 0.05 | 0.01 - 0.10 | | 0.022 | |
| HDL (per log mmol/L) | | -0.19 | -0.34 - -0.03 | 0.017 | | -0.09 | -0.29 - 0.11 | | 0.385 | |
| Triglycerides (per log) | | 0.06 | -0.01 - 0.13 | 0.102 | | -0.01 | -0.10 - 0.07 | | 0.757 | |
| NTproBNP (per log U/L) | | -0.03 | -0.07 - 0.00 | 0.076 | | -0.02 | -0.06 - 0.02 | | 0.407 | |
| **Monocytes (%)** | | **Coef.** | **95% CI** | ***P* value** | | **Coef.** | **95% CI** | | ***P* value** | |
| Sex (woman) | | -0.17 | -0.25 - -0.09 | 3.70∙10^-5^ | | -0.18 | -0.26 - -0.09 | | 9.40∙10^-5^ | |
| Age (per 5 years) | | -0.01 | -0.02 – 0.01 | 0.647 | | 0.01 | -0.02 - 0.02 | | 0.945 | |
| Hypertension | | 0.03 | -0.05 – 0.11 | 0.415 | | 0.08 | 0.00 – 0.16 | | 0.043 | |
| Hba1c (per inverse mmol/L) | | 1.92 | -0.68 - 4.51 | 0.148 | | 1.52 | -1.08 - 4.13 | | 0.250 | |
| HDL (per log mmol/L) | | -0.09 | -0.22 - 0.04 | 0.165 | | 0.01 | -0.01 - 0.15 | | 0.954 | |
| Triglycerides (per log mmol/L) | | 0.04 | -0.02 – 0.10 | 0.158 | | 0.01 | -0.06 - 0.07 | | 0.880 | |
| NTproBNP (per log U/L) | | -0.03 | -0.06 - 0.00 | 0.060 | | -0.02 | -0.05 - 0.01 | | 0.286 | |
| **LMR** | | **Coef.** | **95% CI** | ***P* value** | | **Coef.** | **95% CI** | | ***P* value** | |
| Sex (woman) | | 0.32 | 0.19 – 0.45 | 1.59∙10^-6^ | | 0.39 | 0.26 – 0.53 | | 1.01∙10^-8^ | |
| Age (per 5 years) | | -0.01 | -0.03 – 0.02 | 0.703 | | -0.01 | -0.03 – 0.02 | | 0.969 | |
| Hypertension | | 0.10 | -0.03 – 0.22 | 0.122 | | 0.02 | -0.10 – 0.14 | | 0.742 | |
| Hba1c (per inverse mmol/L) | | -3.24 | -7.39 - 0.91 | 0.126 | | -1.38 | -5.30 - 2.54 | | 0.488 | |
| Triglycerides (per log) | | 0.18 | 0.08 – 0.26 | 2.99∙10^-4^ | | 0.23 | 0.14 – 0.32 | | 9.03∙10^-7^ | |

Coef.: Coefficient, CI: Confidence interval, CRP: C-reactive protein, HDL: High-density lipoprotein, HbA1c: Glycated hemoglobin, NT-proBNP: N-terminal pro brain natriuretic peptide.

**Supplementary Table 5. Outcome at one year follow-up (GIPS III trial)**

| Variable | Women (N=95) | Men (N=284) | *P* value |
| --- | --- | --- | --- |
| No reflow post PCI | 0 (0%) | 0 (0%) | 1.000 |
| Re PCI | 3 (3.2%) | 13 (4.6%) | 0.770 |
| Re ACS | 1 (1.1%) | 6 (2.1%) | 0.658 |
| Death | 0 (0%) | 0 (0%) | 1.000 |

ACS: Acute coronary syndrome, PCI: Percutaneous coronary intervention

**Supplementary Table 6. LMR as a predictor on outcome (GIPS III trial)**

|  | Univariate regression | | | Multivariate regression* | | |
| --- | --- | --- | --- | --- | --- | --- |
| Variable | Coef./OR/HR | 95% CI | *P* value | Coef./OR/HR | 95% CI | *P* value |
| Peak CK, log U/L | -0.33 | -0.60 – 0.06 | 0.019 | -0.31 | -0.60 - -0.03 | 0.032 |
| Peak CK-MB, log U/L | -0.28 | -0.52 - 0.03 | 0.027 | -0.27 | -0.53 - -0.01 | 0.039 |
| Peak Troponin T, log U/L | -0.27 | -0.60 - 0.05 | 0.098 | -0.29 | -0.63 - 0.04 | 0.086 |
| No TIMI reflow post-PCI | NA | NA | NA | NA | NA | NA |
| Infarct size (4 months), % | -0.03 | -0.05 - -0.01 | 0.026 | -0.026 | -0.05 - -0.01 | 0.025 |
| LVEF (4 months), % | 1.28 | -1.07 – 3.64 | 0.284 | 1.04 | -1.40 – 3.49 | 0.402 |
| Re- PCI (1 year) | 1.20 | 0.37 – 3.86 | 0.458 | 1.27 | 0.38 – 4.25 | 0.700 |
| Re-ACS (1 year) | 0.30 | 0.07 – 1.29 | 0.104 | 0.28 | 0.06 – 1.26 | 0.098 |
| Death (1 year) | NA | NA | NA | NA | NA | NA |

Coef: Coefficient, CI: Confidence interval, OR: Odds ratio, HR: Hazard ratio, ACS: Acute coronary syndrome, CK: Creatine kinase, CK-MB: Creatine kinase myocardial band, LMR: lymphocyte to monocyte ratio, PCI: Percutaneous coronary intervention, LVEF: left ventricular ejection fraction. *After adjustment for age and sex.
